# Development of Classification Models for Identifying “True” P-glycoprotein (P-gp) Inhibitors Through Inhibition, ATPase Activation and Monolayer Efflux Assays

**DOI:** 10.3390/ijms13066924

**Published:** 2012-06-07

**Authors:** Simona Rapposelli, Alessio Coi, Marcello Imbriani, Anna Maria Bianucci

**Affiliations:** 1 Department of Pharmaceutical Sciences, University of Pisa, Via Bonanno 6, Pisa 56126, Italy; E-Mail: simona.rapposelli@farm.unipi.it; 2 Consorzio Interuniversitario Nazionale per la Scienza e la Tecnologia dei Materiali (INSTM), Via Giusti 9, Firenze 50121, Italy; E-Mail: alec@farm.unipi.it; 3 Fondazione S. Maugeri, Via S. Maugeri 4, Pavia 27100, Italy; E-Mail: mimbriani@fsm.it; 4 International Centre for Studies and Research in Biomedicine (ICB) A.s.b.l., Luxembourg L-4947, Luxembourg

**Keywords:** P-glicoprotein, decision trees, classification model, consensus model, P-gp inhibitors, MDR1 ligands

## Abstract

P-glycoprotein (P-gp) is an efflux pump involved in the protection of tissues of several organs by influencing xenobiotic disposition. P-gp plays a key role in multidrug resistance and in the progression of many neurodegenerative diseases. The development of new and more effective therapeutics targeting P-gp thus represents an intriguing challenge in drug discovery. P-gp inhibition may be considered as a valid approach to improve drug bioavailability as well as to overcome drug resistance to many kinds of tumours characterized by the over-expression of this protein. This study aims to develop classification models from a unique dataset of 59 compounds for which there were homogeneous experimental data on P-gp inhibition, ATPase activation and monolayer efflux. For each experiment, the dataset was split into a training and a test set comprising 39 and 20 molecules, respectively. Rational splitting was accomplished using a sphere-exclusion type algorithm. After a two-step (internal/external) validation, the best-performing classification models were used in a consensus predicting task for the identification of compounds named as “true” P-gp inhibitors, *i.e.*, molecules able to inhibit P-gp without being effluxed by P-gp itself and simultaneously unable to activate the ATPase function.

## 1. Introduction

P-glycoprotein (P-gp), also known as MDR1, is an ATP-dependent drug efflux pump of 170 kD. It is a member of the ABC superfamily and is abundantly expressed in multidrug resistance (MDR) cells and produced by the *ABCB1* gene [1]. This efflux pump is involved in the protection of tissues of several critical organs. It is highly and normally expressed in the liver, intestine, kidney, brain and placenta, thus influencing xenobiotic disposition. Consequently, P-gp appears to be an important target for the development of new and more effective therapeutics. P-gp plays an important role in multidrug resistance to several cytostatic agents [2–5]; in addition, it seems to be involved not only in limiting the penetration of many exogenous agents across the blood brain barrier (BBB), but also in the aetiology of some neurological disorders [6–10].

As P-gp is a significant component of the BBB, it limits or prevents the input of several chemotherapeutical agents, small peptides, antibiotics, HIV protease inhibitors and antidepressant drugs in the central nervous system (CNS). Its high and homogeneous distribution in the CNS suggests that this kind of efflux pump may be essential both for brain detoxification and for protection against xenobiotics.

The unexpected reduced permeability through the BBB of several highly lipophilic xenobiotics and/or anticancer drugs such as vincristine and doxorubicin may be attributable to the expression of P-gp. P-gp pumps several drugs out of the brain capillary endothelial cells, such as doxorubicin, vincristine and cyclosporin A, thus limiting the accumulation of these molecules within the endothelial cells. On the one hand, this results in the protection of the brain from toxic substances. However, it may represent the main limiting factor in the reduced effectiveness of some therapies in the treatment of neurodegenerative diseases (*i.e.*, Parkinson’s and Alzheimer’s). Several drugs generally used for the treatment of these disorders include P-gp substrates, and consequently their permeability through the BBB could be dramatically reduced.

P-gp inhibition can thus be considered as a valid approach to improve drug bioavailability in tissues where P-gp is highly expressed. One potential strategy to counteract multidrug resistance is the co-administration of a chemotherapeutic agent with a P-gp inhibitor. Thus, the modulation of P-gp is an important goal in overcoming drug resistance in many kinds of tumours characterized by an over-expression of this protein. On the other hand, the co-administration of some drugs with known P-gp inhibitors could lead to adverse neurotoxic reactions caused by the increased accumulation of drugs in the CNS. This is the case for the first and second generations of P-gp modulators (*i.e.*, verapamil, biricodar), which resulted in unacceptable toxicity necessitating a reduction in chemotherapy doses in clinical trials [11].

Several assays have been used for the identification of P-gp substrates, inhibitors, or both. Inhibition experiments (*i.e.*, calcein-AM (acetomethoxy) assays and [^3^H]-vinblastine transport inhibition assay) and the ATP-ase activation assay are commonly used to identify compounds that inhibit the transport of known substrates and to gain info regarding pump activation. In addition, the monolayer efflux assay, which enables the ratio of basolateral-to-apical (B→A) permeability versus apical-to-basolateral (A→B) permeability to be calculated, can be useful for identifying P-gp substrates [12].

Our study was carried out using a unique dataset of 59 compounds for which there were data referring to inhibition, ATPase activation and monolayer efflux assays. The aim was to develop classification models (CMs) to identify “true” P-gp inhibitors. We define them as being “true” in the sense that they are compounds that are able to inhibit P-gp, without being effluxed by P-gp itself and simultaneously unable to activate the ATPase function. We originally introduced this definition [13] based on criteria suggested by Polli *et al.* [12]. Applying this hypothesis, the simultaneous use of the three types of classification models could help to identify new chemical entities according to the definitions summarized in Table 1.

A “true” P-gp inhibitor is positive only in the inhibition assay (it is not effluxed, nor does it activate the pump); a substrate is positive in all three assays (it activates the pump, it is effluxed, and inhibits the transport of a reference substrate, probably with a competitive mechanism); a non-substrate is negative in all three assays.

Regarding the specific assays used to label the compounds, the calcein AM assay was used to assess whether or not a compound acts as a P-gp inhibitor. The calcein fluorescence is measured in a relative fluorescence unit (RFU). The percentage maximum response is defined by dividing the test compound’s RFU response by the elacridar (a potent, specific Pgp inhibitor) RFU response.

The drug-stimulated ATPase activity (nmol/min/mg of protein) is determined as the difference between the amounts of inorganic phosphate released from ATP in the absence and presence of vanadate. Drug-stimulated Pgp ATPase activity is reported as fold-stimulation in relation to the basal Pgp ATPase activity in the absence of drug.

Regarding the monolayer efflux assay, the ratio of the B→A/A→B apparent permeability (*P*_app_) values is calculated. The involvement of a Pgp-mediated efflux mechanism (class Y) is suggested when the B→A/A→B ratio is ≥2.1.

The predictive power of the developed CMs was estimated using a test set selected for each experiment from the initial dataset of 59 compounds. The best-performing models evaluated in terms of robustness and predictivity, obtained within each experiment, were used as a consensus model for a further predictive task performed on an additional external set of compounds already synthesized by us [13–16]. All models were validated through currently accepted criteria for statistical analysis [17].

## 2. Results and Discussion

### 2.1. Leave-One-Out cross Validation and Test Set Prediction

Several classification models were built by using random tree (RT) and C4.5 algorithms and, for each model, specific parameters were changed, as previously described.

Leave-One-Out Cross-Validation (LOO-CV) was used to estimate the classification results on the training sets of each experiment: on the basis of the computed statistical parameters mentioned in Section 3.3, only CMs with an accuracy ≥ 70%, Matthews Correlation Coefficient (MCC) ≥ 0.40, K ≥ 0.40 and Area Under the ROC Curve (AUC) ≥ 0.60 were selected. Tables 2–4 report the statistical parameters such as sensitivity (True Positives, TP), specificity (True Negatives, TN), accuracy, MCC, K statistic, and AUC obtained from LOO-CV and from the prediction on the test sets for the best decision trees developed in the three experiments. At least one model developed with the C4.5 algorithm was reported for each experiment, even when statistics did not satisfy the requested criteria.

Looking at the statistical parameters (in particular, accuracy, MCC and K statistic) obtained from LOO-CV, the models are expected to possess a good predictive power; however, a second validation step was carried out on a test set selected for each experiment (see Section 3.1) in order to support this result. All compounds of the test set belong to the applicability domains (AD) defined by the training set of each experiment (see Section 3.4 for a definition of AD).

With regard to the inhibition experiment, 19 molecular descriptors were used for developing the models. After the two-step validation, three best-performing decision trees (RT method) were selected— see Figure S1 in Supporting Information for their graphical representation.

The **RT(S10 K11)** model produced the best predictions for the classification of the P-gp inhibition (Table 2), showing the highest level of similarity between the internal LOO-CV, with a TP of 68.8 and TN of 82.6%, and the external test set, with a TP of 60 and TN of 80%. **RT(S10 K11)** also showed the highest MCC, K and AUC compared to the other classification models for P-gp inhibition. On the other hand, C4.5 showed the lowest values for each parameter in the internal LOO-CV and external validation on the test set, in comparison with the other models referring to the P-gp inhibition experiment.

**RT(S9 K10)** was found by randomly choosing a maximum of 10 molecular descriptors (K10) at each node; six molecular descriptors were involved in the model (Table 5). **RT(S10 K11)** and **RT(S1 K11)** were found by randomly choosing a maximum of 11 molecular descriptors at each node; both models were finally based on five molecular descriptors.

Only seven of the initially exploited 19 molecular descriptors were involved in these decision trees: XLogP, molar refractivity (AMR), number of single bonds, excluding bonds with hydrogen atoms and aromatic bonds (nBondsS3), and Ghose-Crippen LogK_ow_ (ALogP) was common to all models (Table 5). Topological Polar Surface Area (TopoPSA) was common to models **RT(S9 K10)** and **RT(S10 K11)**. The SMARTS pattern: O:C-C:O (PubchemFP544) and a doubly bound carbon, bound to another carbon, = C–C– (C1SP2) were only involved in **RT(S1 K11)** and **RT(S9 K10)**, respectively.

XLogP created the highest separation (13/4) of the Y/N compounds; most of the inhibitors had a XLogP ≥ 1.6 (13/16 Y compounds, as observed in **RT(S9 K10)**); a topological polar surface area > 23.76 Å^2^, and a Ghose-Crippen LogK_ow_ (ALogP) ≥ −0.76 (12/16 class Y compounds, as observed in **RT(S9 K10)**). Having a high PSA corresponds to a high proportion of electronegative elements (*i.e.*, nitrogen and oxygen atoms) and accounts for the poor penetration of molecules in a hydrophobic environment (*i.e.*, biological membranes). However, it may account for their easy penetration in hydrophilic environments, such as the core of transporter proteins. Generally, molecules with a PSA > 140 Å^2^ are believed to have a low capacity for penetrating cell membranes, while those with PSA < 60 Å^2^ are easily absorbed [18]. Our finding is in agreement with Gadhe *et al.* [19] who carried out a CoMFA and HQSAR study, highlighting the importance of the presence of electronegative elements for a compound to be an inhibitor. Of the inhibitors belonging to our training set and characterized by a high proportion of electronegative atoms, nitrendipine, nicardipine and nifedipine are examples of compounds bearing a nitro group. This aspect also was also observed by Gadhe *et al.* who found that a nitro group (together with methoxy and ether) can lead to a good inhibitory potency.

For the ATPase activation experiment, 18 molecular descriptors were used for developing the models. After LOO-CV and the prediction task on the test set, three best-performing decision tree models (RT method) were selected—see Figure S2 in Supporting Information for their schematic representation.

The **RT(S5 K3)** and **RT(S10 K2)** models produced the best predictions for the classification of the ATPase activation experiment (Table 3). The **RT(S5 K3)** and **RT(S10 K2)** models showed the best similarity between the internal LOO-CV, with a TP of 84.2 and 73.7% and a TN of 80%, and the external test set, with a TP of 80 and 60% and TN of 60 and 80%, respectively. **RT(S5 K3)** showed the highest MCC, K and AUC, compared to the other classification models for the ATPase activation experiment. Unlike the models developed with the RT algorithm, C4.5 showed the lowest values for each parameter in the external test set.

**RT(S10 K2)**, **RT(S5 K3)** and **RT(S10000 K8)** were found by randomly choosing a maximum of 2, 3 and 8 molecular descriptors at each node; the models were based on 9, 10, and 7 descriptors, respectively (Table 5).

Finally, of the 18 descriptors exploited in developing the models, only 13 were involved in these decision trees and six are common to all the selected decision trees (Table 5): topological polar surface area (TopoPSA), excessive molar refraction (MLFER_E), number of six-membered rings (n6Ring), number of H-bond donors (nHBDon), number of H-bond acceptors (nHBAcc), and number of six membered rings, including the fused ones (nT6Ring). Two molecular descriptors, number of rings (nRing) and heteroaromatic rings (PubchemFP256), were common to **RT(S5 K3)** and **RT(S10 K2)**. The detailed atom neighbourhood pattern C(−C)(−N)(=C) (PubchemFP437) and the simple SMARTS pattern N–C–C–C–C (PubchemFP592) were involved in **RT(S5 K3)**. The simple SMARTS patterns C–N–C:C and N–C–C–C:C) (Pubchem FP495 and PubchemFP607) were involved in **RT(S10 K2)**. Finally, the simple atom nearest neighbour pattern N(~C) (~C) (~H) (PubchemFP392) was only present in **RT(S10000 K8)**.

Looking at **RT(S10 K2)** (Figure S2 in Supporting Information), we observe that all the considered ATPase activators have a polar surface area higher than 29.77 Å^2^. This is in agreement with Fernandes and Gattass, who analyzed the chemical properties of some known P-gp substrates and their results supported the hypothesis that P-gp pumps out substrates with a high TopoPSA [20].

Looking at the decision trees for the ATPase activation experiment (Figure 2 in Supporting Information), highlights that although the trees do not indicate a clear separation between activators and non-activators: in general, most of the ATPase activators have a number of H-bond donors (nHBDon) < 3 and a number of H-bond acceptors (nHBAcc) < 9, an excessive molar refraction (MLFER_E) ranging between 1.92 and 3.64 and they have at least one the N–C–C–C:C pattern (where “:” refers to an aromatic bond) (PubchemFP607). Furthermore, most of the non-activators have more than two six-membered rings (n6Ring).

For the monolayer efflux experiment, 22 molecular descriptors were used to develop the models. After the two-step statistical validation, five best-performing decision tree models (RT method) were selected—see Figure S3 in Supporting Information for their graphical representation.

The **RT(S20 K4)** model produced the best predictions for the classification of the monolayer efflux experiment (Table 4). This model also showed the highest level of similarity between the internal LOO-CV, with a TP of 83.3 and a TN of 66.7%, and the external test set, with a TP of 80 and TN of 60%. Moreover, **RT(S20 K4)** showed the highest MCC, K and AUC, compared to the other classification models for the monolayer efflux experiment. On the other hand, C4.5 showed the lowest values for each parameter in the internal LOO-CV and external test set, compared to each model in the monolayer efflux experiment.

**RT(S20 K4)** and **RT(S30 K4)** were constructed by randomly choosing a maximum of four molecular descriptors at each node. **RT(S80 K14)** and **RT(S1000 K14)** were obtained by choosing the optimal descriptors from the 14 descriptors available, and **RT(S80 K15)** was constructed by randomly choosing a maximum of 15 molecular descriptors at each node. The final models were based on 9, 10, 8, 6, and 7 descriptors, respectively (Table 5).

Lastly, only 14 of the exploited 22 molecular descriptors were involved in these decision trees: the number of single bonds, excluding bonds with hydrogen atoms and aromatic bonds (nBondsS3) and excessive molar refraction (MLFER_E), were common to all five decision trees (Table 5). XLogP, a double bond C atom bound to three other C atoms (C3SP2), the overall summation solute H-bond acidity (MLFER_A), and Ghose-Crippen LogK_ow_ (ALogP), were common to four out of five models. Molar refractivity (AMR) was involved in **RT(S80 K15)**, **RT(S20 K4)**, and **RT(S80 K14)**; double bond C atom bound to two other C atoms (C2SP2), number of H-bond acceptors (nHBAcc), and number of rings (nRing) were common to two out of five decision trees. Finally, Mannhold LogP (MLogP) and simple atom pair N-H (PubchemFP299), count of heterocycles (SubFPC275), single bound C atom bound to two other C atoms (C2SP3) and complex SMARTS pattern Cc1cc(N)ccc1 (PubchemFP737) were only involved in **RT(S20 K4)**, **RT(S80 K15)**, and **RT(S30 K4)**, respectively.

The graphical representation of the decision trees (Figure S3 in Supporting Information) highlights that 21 out of 24 of the effluxed compounds have at least 12 single bonds (excluding bonds with hydrogens and aromatic bonds) (nBondsS3), nBondsS3 molecular descriptor also marks the highest separation (22/6) among effluxed(Y)/non-effluxed(N) compounds. A total of 23 out of 24 effluxed compounds have a MLFER_E ≥ 1.52, and less than six double bound C atoms bound to three other C atoms (C3SP2). At least 20 of the 24 effluxed compounds have a Mannhold LogP (MLogP) ≥ 2.51, and almost 17/24 have almost 3 single bound C atom bound to two other C atoms (C2SP3), almost a simple atom pair N-H (PubchemFP299), less than five heterocycles (SubFPC275) and less than five rings (nRing).

In summary, the molecular descriptors characterizing the best-performing classification models appear to be involved (within each experiment) in different trees showing comparable performances. This can be considered as a proof of the stability and reliability of the models, and suggests that the performance depends on the sets of molecular descriptors chosen for each experiment (the selection of descriptors is described in Section 3.1).

Furthermore, detailed analysis of the decision trees in the three experiments suggested a pool of molecular features that can help in the development of new chemical entities potentially endowed with a profile typical of a “true” inhibitor. Some of them, (e.g., polar surface area and LogP for the inhibition experiment and nBondsS3 for the efflux experiment), gave quite a clear separation between Y/N compounds.

### 2.2. Consensus Modelling and Prediction Task on an Additional External Set

Since all the models were reasonably accurate with regard to the prediction on the test set compounds, a consensus model was built for each experiment, by considering the predictions and taking into account the applicability domain of each individual model. The statistical parameters for LOO-cross validation and for the predictive task on the test sets were calculated using a majority voting criteria (*i.e.*, when the majority of models constituting the consensus model classify a compound as Y (or N), the prediction output of the consensus model is Y (or N)); results are reported in Table 6.

The results showed that the prediction accuracies of the consensus models, considering the statistical parameters obtained for both the LOO-CV and for the prediction task on test sets, were generally better than those of any individual model. This is highlighted in the inhibition experiment where the best models (**RT(S9 K10**) and **RT(S10 K11)**) showed an accuracy of 76.9% and a MCC = 0.51–0.52 on LOO-CV and an accuracy on the test set of 70%. The consensus model shows an improved accuracy for both LOO-CV and the test set prediction (84.6 and 80%, respectively) and a MCC = 0.67.

The consensus models were tested on the external set constituted by compounds that we had previously synthesized and tested in order to further evaluate the power of the consensus prediction. All the external set compounds belong to the applicability domain defined by each training set constituting the consensus models. Results of the prediction task are reported in Table 7.

The overall accuracy is similar to that observed on the test sets and comparable to that found by other studies on P-gp substrates [21,22].

As observed in Section 2.1, only two compounds of this additional external set, II_14a and II_15a, are considered effective “true” inhibitors; since they inhibit P-gp with a higher power than elacridar, they do not activate ATPase, nor are they effluxed by P-gp. The consensus models correctly predict these two compounds and the descriptor nBondsS3 appear to be determinant in identifying them, in particular among the compounds of series “III”: II_14a and II_15a have less than 12 single bonds (excluding bonds with H atoms and aromatic bonds). In addition, II_11b, II_13a, II_13b, II_16a, II_17a, II_17b and II-23 have nBondsS3 < 12, however, they are discarded by the consensus models as “true” inhibitors probably because of their polar surface area which is not in the ideal range (23.76–29.77 Å^2^) for a compound that can inhibit P-gp without activating ATP-ase.

## 3. Experimental Section

### 3.1. Dataset and Molecular Descriptors

The classification models were developed and validated using biological data referring to 59 compounds taken from Polli *et al.* [12] belonging to heterogeneous chemical classes and for which homogeneous biological data referring to inhibition, ATPase activation and monolayer efflux assays were available (Table 8).

Of the 59 selected compounds, 26 act as inhibitors (class Y) and 33 do not (class N); 29 compounds stimulate ATPase activity (class Y) and 30 are not ATPase activators (class N); 34 compounds are effluxed (B→A/A→B ratio > 2.1) and 25 do not undergo efflux. Of these, only elacridar, GW420867, and testosterone are considered to be “true”-inhibitors (YNN profile), with elacridar being the most potent. This binary classification was performed according to thresholds defined for each assay [12].

For the P-gp inhibition experiment, in the calcein AM assay the percentage maximum response is defined by dividing the test compound’s RFU response by the elacridar RFU response. All compounds with a response < 10% of the maximum were labelled as negative in the assay (class N). Vinorelbine, vincristine, and propranolol had a maximum response between 6% and 12%, thus belonging to the non-confident zone, a region where it is difficult to classify a compound as “Y” or “N”. However, since these three compounds had a response <10% of maximum, they were labelled as “N” [12].

Drug-stimulated Pgp ATPase activity was reported as fold-stimulation in relation to the basal Pgp ATPase activity in the absence of drugs (DMSO control). A compound was classified as an ATPase activator (class Y) if the fold-stimulation was > 2 over the DMSO control. Daunorubicin fell into the non-confident range of ATPase stimulation (between 1.5 and 2.0); however, because the stimulation ratio was below 2-fold, the compound was labelled as “N” [12].

Regarding the monolayer efflux assay, a Pgp-mediated efflux mechanism (class Y) is involved if the B→A/A→B ratio is ≥ 2.1. For compounds with B→A/A→B ratios between 1.5 and 2.0, a follow-up experiment with 2 μM elacridar was completed to confirm that the compound was effluxed [12].

In this study, aimed at developing classification models for the identification of “true” P-gp inhibitors, a unique dataset was exploited for which homogeneous biological data of inhibition, ATPase activation and efflux assays were available. We used an initial dataset containing biological data characterized by the required homogeneity but, at the same time, characterized by low variability. In fact, the data came from the same research group and were acquired by using the same experimental protocol, which ensures that reliable models are obtained. Although the limited size of the dataset may lead to the development of models characterized by a limited applicability domain, the creation of a consensus model for each type of experiment should help to overcome this problem. The overall coverage of chemical space afforded by the consensus model is expected to be high, because it is rare to have an unknown compound outside of the defined applicability domain of all available models that constitute the consensus model.

Looking at the number of compounds for each class, it can be noted that the three datasets are quite well balanced, which is a critical requirement for the development of a classification model [23].

The predictive power of the classification models was also tested on an external set made up of 47 derivatives which we had already synthesized and tested (Table 9) [13–16].

All 47 selected compounds were tested for inhibition, ATPase activation and monolayer efflux, and were labelled as Y/N. In terms of the inhibition assay, since almost all the synthesized compounds (except III_7a and MV181) are labelled as inhibitors with a potency higher than verapamil, seven non-inhibitors (I_5a–b, I_6a–b, I_7a–b, and I_8a) from reference [14] were added in order to increase the N class, and to better assess the classification power of the models on our synthesized compounds. A total of 26 out of 45 inhibitors were stronger than elacridar, of which only two (II_14a, II_15a) are considered as “true” inhibitors, since they do not activate ATPase and are not effluxed by P-gp.

Molecular descriptors were calculated by PaDEL, a software that enables the computation of 2D and 3D descriptors and several types of fingerprints [24]. A total of 696 molecular descriptors and Pubchem fingerprints and substructure fingerprints [25] were calculated for compounds of both the modelling and the external set (Table 10).

For each experiment, the dataset was split into a training set of 39 molecules and a test set of 20. Test sets were rationally selected using the calculated molecular descriptors. Each molecule of the initial dataset was represented as a point in a multi-dimensional space defined by all the descriptors. The dataset was thus split into a training/test set pair for each experiment, so that points representing both training and test sets were distributed within the whole descriptor space occupied by the entire dataset, and each point of the test set was close to at least one point of the training set. This ensures that the similarity principle is followed when activity is predicted on the test set. Rational splitting was accomplished using a Sphere-Exclusion type algorithm [26], which we had subsequently optimized [27]. However, the selection was “driven” so that the two classes of each experiment were equally represented in the test set (10 compounds for each class Y/N) as commonly suggested for binary classification models [23]. The compounds selected for each test set are shown in Table 11.

For each experiment, the initial high number of descriptors was pre-selected, which enabled us to leave out of the subsequent modelling process any descriptors that do not change significantly across the molecules of the dataset or those that were too strictly correlated (*i.e.*, that do not contain the desired degree of information). Descriptors with constant values and those showing an inter-correlation higher than 0.95 were thus discarded.

After that, in order to determine the right combination of molecular descriptors to be used in the search for good models, the CfsSubsetEval attribute evaluator [28] within the WEKA software [29], was applied and a genetic algorithm was chosen as the search method. This method evaluates the worth of a subset of attributes by considering the individual predictive ability of each feature along with the degree of redundancy between them. Subsets of features that are highly correlated with the class of compound while having low inter-correlation are preferred. Using a Leave-One-Out (LOO) cross-validation on each training set, and performing 10 runs with different seed numbers to obtain averaged results, subsets of molecular descriptors were selected according to the average percentage of a ranking value. Following this method, the molecular descriptors were reduced to three subsets of 19, 18, and 22 descriptors which were used to develop classification models for the inhibition, ATPase activation and monolayer efflux experiments, respectively.

### 3.2. Classification Methods

Decision trees are one of the most widely used forms of machine learning enabling data mining for predictive purposes. In this study, Random tree and C4.5 [30] algorithms available in WEKA were used to develop predictive CMs.

The Random Tree algorithm builds a tree that considers K randomly chosen attributes at each node. Models were developed by changing this parameter between 1 and the maximum number of molecular descriptors used for model development (19, 18, and 22 descriptors for the inhibition, ATPase activation, and monolayer efflux experiments, respectively). Thus, no more than 10 descriptors (with a minimum number of 5) were involved in the best-performing decision trees (Table 5). This number of descriptors appears to be suitable for the size of the dataset exploited when using classifications approaches. No maximum depth for the tree was fixed. The minimum total weight of the instances in a leaf was set to 1 and 20 different random seeds were used.

In C4.5, pruning was used by setting the confidence factor to 0.25 (default value). The minimum number of instances (compounds) per leaf varied from 1 to 5. The amount of data (NumFolds) for reduced-error pruning was also varied.

In all cases, we tried to obtain statistically validated decision tree models that were as small as possible and that had large leaf nodes. This would then lead to models that were probably easier to interpret and that had a better predictive power (e.g., a leaf with 100 compounds in it will have more predictive power than one with just one or two compounds).

### 3.3. Statistical Validation

Parameters needed for model validation were computed and analyzed for the validation step for both the training (internal validation) and the selected test set (external validation).

Leave-one-out cross validation on the training sets was employed for the internal validation and to assess the robustness of the model. Models were estimated on the basis of true positives (TP), or sensitivity (fraction of “class Y” molecules correctly classified), true negatives (TN) or specificity, (fraction of “class N” molecules correctly classified), and accuracy (fraction of molecules correctly classified) on the Training set. Other statistics were considered, such as the Matthews Correlation Coefficient (MCC) [31], the *K* statistic [32], and the area under the Receiver Operating Characteristic (ROC) curve (AUC) [33].

The MCC is a measure of the quality of classification. It is expressed by values ranging between −1 and +1, where +1 represents a perfect prediction, 0 an average random prediction, and −1 means an inverse prediction. The MCC is considered one of the best parameters to estimate what is reported in the so-called *confusion matrix* with regard to true and false positives and negatives. The equation used for computing MCC values is the following:

$$MCC=\frac{\left(TP\times TN\right)-\left(FP\times FN\right)}{\sqrt{\left(TP+FP\right)\left(TP+FN\right)\left(TN+FP\right)\left(TN+FN\right)}}$$

where TP is the number of true positives, TN the number of true negatives, FP the number of false positives (“class N” compounds predicted as “class Y”), and FN the number of false negatives (“class Y” compounds predicted as “class N”). The advantage of the MCC is that it can be used to evaluate the quality of classification models even when developed on an unbalanced dataset.

*K* is the chance-corrected proportional agreement: it is an index that compares the agreement found versus the agreement that might be expected by chance. *K* = 1 corresponds to a perfect agreement, *K* = 0 means an agreement equal to what expected by chance, and *K* = −1 means complete disagreement.

The ROC curve can be represented by plotting the fraction of TP (or sensitivity) versus the fraction of FP (or 1-specificity). The ROC analysis helps to select optimal classification models and to discard the suboptimal ones. The ROC curve provides a criterion based on the so-called AUC (Area Under the ROC curve), which is an index of goodness of the classification model: a perfect CM shows AUC = 1.

When evaluating the results of a classification model, the reference status is generally considered as the one where all of the objects are assigned to the class that is most represented. This reference condition corresponds to the absence of a model, and is therefore called a *No-model* condition. Statistical parameters similar to those obtained for the *No-model* status provide evidence of poor results from the classification model, as the *No-model* value is unique and does not depend on the classification method used.

### 3.4. Applicability Domain

Several methods to define the Applicability Domain of a QSAR model have been reported in the literature; in this work, we exploited a distance-based method [34]. In this approach, the applicability domain (AD) where the model is expected to give reliable predictions is defined through a similarity-based criterion. This enables us to leave out from the prediction task those compounds whose structural features were poorly sampled in the training set and whose predictions are not reliable.

In order to estimate structural similarity, each compound was represented by a point in the *N*-dimensional descriptor space (where *N* is the total number of descriptors); its coordinates (*X_1_*, *X_2_*, …, *X_N_*, where *X_i_*) take the values of each individual descriptor. The molecular similarity between all the pairs of molecules was measured in terms of the Euclidean distance between their representative points. Compounds with the smallest distance between them have the highest similarity.

In addition, we needed to define a centroid of the training set: this was calculated as a point in the *N*-dimensional descriptors space with coordinates $\bar{X_{1}},\bar{X_{2}},\ldots ,\bar{X_{N}}$, where $\bar{X_{i}}$ is the average value of the *i* descriptor within the training set. The Euclidean distances between the centroid and each of the molecules of the training set were then computed, so that the minimum and maximum distances from the centroid were used to define the AD (allowed range). Test set and external set compounds that have to be subjected to the prediction task must fall within the AD of the model: the distances between each molecule and the centroid must be included within the allowed range.

The consensus model obtained from each experiment was constructed by averaging all available predicted values by taking into account the applicability domain of each individual model.

Since each model had its unique way of defining the AD, each compound of the test set and the external set could be found within the AD of one or more models constituting the consensus model, so only models “covering” the compound were used for averaging. The advantage of this method is that the overall coverage of the prediction is still high since it is rare to have an unknown compound outside of the defined ADs of all available models [23].

## 4. Conclusions

P-gp inhibition may be considered as a valid approach to improve drug bioavailability as well as to overcome the drug resistance to many kind of tumours characterized by an over-expression of this protein. The development of predictive models for identifying “true” P-gp inhibitors could be valuable in selecting new chemical entities that inhibit P-gp without affecting ATPase activity and that are not actively effluxed by P-gp.

Three experiments to develop classification models aimed at identifying “true” P-gp inhibitors were thus performed. The study was carried out using decision tree algorithms on a unique dataset of 59 compounds for which data referring to inhibition, ATPase activation and monolayer efflux assays were available. The predictive power of the models was assessed using a LOO-CV for internal validation and a selected test set for the external validation. This two-step validation led to a pool of best-performing models for each experiment, which were used as a consensus model that showed a better performance than any single model. Furthermore, the consensus models correctly identified the “true” inhibitors from the additional external set of compounds that we had synthesized. The molecular descriptors characterizing the best-performing classification models appeared to be involved (within each experiment) in different trees, thus confirming the stability and reliability of the models. The analysis of the decision trees suggested a pool of molecular features that could help in the development of new chemical entities potentially endowed with the typical profile of a “true”-inhibitor.

## Supplementary Information



## Figures and Tables

**Table 1:** Summary of definitions for “true” p-glycoprotein (P-gp) inhibitors, P-gp substrates or non-substrates.

Definition	P-gp Inhibition	ATPase activation	Efflux
“True” inhibitor	Y	N	N
Substrate	Y	Y	Y
Non-substrate	N	N	N

Inhibition assay, Y: inhibitor; ATPase activation, Y: activator; efflux, Y: effluxed compound.

**Table 2:** Classification models on P-gp inhibition experiment: leave-one-out (LOO) cross-validation statistical parameters and prediction task on the test set.

Model	LOO-cross validation statistics						Test set statistics		
	
	TP	TN	Acc	MCC	K	AUC	TP	TN	Acc
RT(S9 K10)	62.5	87.0	76.9	0.51	0.51	0.75	90.0	50.0	70.0
RT(S10 K11)	68.8	82.6	76.9	0.52	0.52	0.76	60.0	80.0	70.0
RT(S1 K11)	68.8	78.3	74.4	0.47	0.47	0.74	90.0	70.0	80.0
C4.5	37.5	65.2	53.8	0.19	0.48	0.52	60.0	50.0	55.0

TP: True positives (sensitivity for inhibitor, class Y); TN: true negatives (specificity for class N); Acc: Accuracy; MCC: Matthews correlation coefficient; K: K statistic; AUC: Area Under the ROC Curve; S: seed number; K: number of randomly chosen molecular descriptors at each node.

**Table 3:** Classification models on ATPase activation experiment: LOO cross-validation statistical parameters and prediction task on the test set.

Model	LOO-cross validation statistics						Test set statistics		
	
	TP	TN	Acc	MCC	K	AUC	TP	TN	Acc
RT(S5 K3)	84.2	80	82.1	0.64	0.64	0.82	80.0	60.0	70.0
RT(S10 K2)	73.7	80	76.9	0.54	0.54	0.77	60.0	80.0	70.0
RT(S10000 K8)	73.7	70	71.8	0.44	0.44	0.72	70.0	80.0	75.0
C4.5	89.5	75	82.1	0.65	0.64	0.86	60.0	50.0	55.0

TP: True positives (sensitivity for ATPase activator, class Y); TN: true negatives (specificity for class N); Acc: Accuracy; MCC: Matthews correlation coefficient; K: K statistic; AUC: Area Under the ROC Curve; S: seed number; K: number of randomly chosen molecular descriptors at each node.

**Table 4:** Classification models on monolayer efflux experiment: LOO cross-validation statistical parameters and prediction task on the test set.

Model	LOO-cross validation statistics						Test set statistics		
	
	TP	TN	Acc	MCC	K	AUC	TP	TN	Acc
RT(S80 K15)	79.2	60	71.8	0.40	0.40	0.70	80.0	70.0	75.0
RT(S20 K4)	83.3	66.7	76.9	0.51	0.51	0.75	80.0	60.0	70.0
RT(S80 K14)	83.3	60	74.4	0.45	0.44	0.72	70.0	60.0	65.0
RT(S30 K4)	70.3	73.3	71.8	0.44	0.43	0.72	80.0	50.0	65.0
RT(S1000 K14)	79.2	66.7	74.4	0.46	0.46	0.73	60.0	70.0	65.0
C4.5	75.0	40.0	61.5	0.16	0.16	0.41	100.0	40.0	70.0

TP: True positives (sensitivity for effluxed compounds, class Y); TN: true negatives (specificity for class N); Acc: Accuracy; MCC: Matthews correlation coefficient; K: K statistic; AUC: Area Under the ROC Curve; S: seed number; K: number of randomly chosen molecular descriptors at each node.

**Table 5:** Number and type of molecular descriptors involved in each model developed for the inhibition, ATPase activation, and monolayer efflux experiments.

Inhibition	
**Model**	***n*° of Molecular Descriptors involved**
RT(S9 K10)	6
RT(S10 K11)	5
RT(S1 K11)	5
**Molecular descriptor**	***n*° of models in which the descriptor is involved**
XLogP	3
AMR	3
nBondsS3	3
Ghose-Crippen LogK_ow_	3
TopoPSA	2
PubchemFP544	1
C1SP2	1

**ATPase activation**	

**Model**	***n*° of Molecular Descriptors involved**
RT(S5 K3)	10
RT(S10 K2)	9
RT(S10000 K8)	7
**Molecular descriptor**	***n*° of models in which the descriptor is involved**
TopoPSA	3
MLFER_E	3
n6Ring	3
nHBDon	3
nHBAcc	3
nT6Ring	3
nRing	2
PubchemFP256	2
PubchemFP392	1
Pubchem FP437	1
PubchemFP495	1
PubchemFP592	1
PubchemFP607	1

**Monolayer efflux**	

**Model**	***n*° of Molecular Descriptors involved**
RT(S30 K4)	10
RT(S20 K4)	9
RT(S80 K14)	8
RT(S80 K15)	7
RT(S1000 K14)	6
**Molecular descriptor**	***n*° of models in which the descriptor is involved**
nBondsS3	5
MLFER_E	5
XLogP	4
C3SP2	4
MLFER_A	4
Ghose-Crippen LogK_ow_	4
AMR	3
C2SP2	2
nHBAcc	2
nRing	2
Mannhold LogP	1
C2SP3	1
PubchemFP299	1
PubchemFP737	1
SubFPC275	1

**Table 6:** Consensus models for inhibition, ATP-ase activation and monolayer efflux experiments.

	LOO-cross validation statistics						Test set statistics		
		
Model	TP (Y)	TN (N)	Acc	MCC	K	AUC	TP (Y)	TN (N)	Acc
Inhibition	68.8	95.7	84.6	0.67	0.67	0.82	90.0	70.0	80.0
ATPase activation	78.9	75	76.9	0.54	0.54	0.84	70.0	80.0	75.0
Monolayer efflux	83.3	66.7	76.9	0.44	0.51	0.75	80.0	70.0	75.0

TP: True positives; TN: true negatives; Acc: Accuracy; MCC: Matthews correlation coefficient; K: K statistic; AUC: Area Under the ROC Curve; S: seed number; K: number of randomly chosen molecular descriptors at each node.

**Table 7:** Consensus models statistics for the prediction task on the additional external set.

	External set statistics		

Model	TP (Y)	TN (N)	Accuracy
Inhibition	77.1	83.3	77.8
ATPase activation	75.0	71.4	72.3
Monolayer efflux	74.4	100.0	76.6

TP: True positives; TN: true negatives; Acc: Accuracy; Y: inhibitor, ATPase activator, or effluxed compound.

**Table 8:** Dataset of 59 compounds, with their IAE profile.

Compound	Inhibitor	ATPase activator	Efflux	Compound	Inhibitor	ATPase activator	Efflux
Amantadine	N	N	N	Testosterone	Y	N	N
Chlorpheniramine	N	N	N	Chlorpromazine	Y	Y	N
Doxorubicin	N	N	N	Ketoconazole	Y	Y	N
Itraconazole	N	N	N	Mebendazole	Y	Y	N
Lidocaine	N	N	N	Midazolam	Y	Y	N
Mannitol	N	N	N	Nicardipine	Y	Y	N
Methotrexate	N	N	N	Nifedipine	Y	Y	N
Practolol	N	N	N	Nitrendipine	Y	Y	N
Propranolol	N	N	N	Verapamil	Y	Y	N
Pyridostigmine	N	N	N	Chloroquine	N	N	Y
Ranitidine	N	N	N	Cimetidine	N	N	Y
Sumatriptan	N	N	N	Colchicine	N	N	Y
Triamterene	N	N	N	Daunorubicin	N	N	Y
Yohimbine	N	N	N	Dexamethasone	N	N	Y
Amprenavir	Y	Y	Y	Etoposide	N	N	Y
Diltiazem	Y	Y	Y	Hoechst 33342	N	N	Y
Dipyridamole	Y	Y	Y	Mitoxantrone	N	N	Y
Loperamide	Y	Y	Y	Neostigmine	N	N	Y
Loratadine	Y	Y	Y	Puromycin	N	N	Y
Monensin	Y	Y	Y	Vincristine	N	N	Y
Nelfinavir	Y	Y	Y	Vinorelbine	N	N	Y
Prazosin	Y	Y	Y	Clarythromycin	N	Y	Y
Quinidine	Y	Y	Y	Eletriptan	N	Y	Y
Reserpine	Y	Y	Y	Emetine	N	Y	Y
Ritonavir	Y	Y	Y	Erythromycin	N	Y	Y
Saquinavir	Y	Y	Y	Indinavir	N	Y	Y
Terfenadine	Y	Y	Y	Taxol	N	Y	Y
Vinblastine	Y	Y	Y	Trimethoprim	N	Y	Y
Elacridar	Y	N	N	Cyclosporin A	Y	N	Y
GW420867	Y	N	N				

**Table 9:** External set of 47 synthesized compounds. Suffix I, II, and III refer to references [13–15] respectively; MV181 is taken from ref [16]. Inhibitors with a potency similar to or higher than elacridar are reported in bold in the second column.

Compound	Inhibitor	ATPase activator	Efflux	Compound	Inhibitor	ATPase activator	Efflux
I_8b	Y	N	Y	III_7c	Y	Y	Y
II_11a	Y	N	N	III_7d	**Y**	Y	Y
II_11b	Y	N	Y	III_8a	Y	Y	Y
II_13a	Y	N	Y	III_8b	Y	Y	Y
II_13b	Y	N	Y	III_8c	**Y**	Y	Y
II_14a	**Y**	N	N	III_8d	Y	Y	Y
II_14b	**Y**	Y	Y	III_9a	**Y**	N	Y
II_15a	**Y**	N	N	III_9b	**Y**	N	Y
II_15b	**Y**	N	Y	III_9c	**Y**	N	Y
II_16a	**Y**	N	Y	III_9d	**Y**	N	Y
II_16b	Y	N	Y	III_10a	**Y**	N	Y
II_17b	**Y**	Y	N	III_10b	Y	N	Y
II_23	**Y**	N	Y	III_10c	**Y**	N	Y
II_25	**Y**	N	Y	III_10d	Y	N	Y
II_26	**Y**	N	Y	III_11a	Y	N	Y
II_27	**Y**	N	Y	III_11b	**Y**	N	Y
III_5a	Y	N	Y	III_11c	**Y**	N	Y
III_5b	Y	N	Y	III_11d	Y	N	Y
III_5c	**Y**	N	Y	III_12a	**Y**	Y	Y
III_5d	Y	N	Y	III_12b	**Y**	N	Y
III_6b	Y	N	Y	III_12c	**Y**	N	Y
III_6c	**Y**	N	Y	III_12d	**Y**	Y	Y
III_7a	N	Y	Y	MV181	N	N	Y
III_7b	Y	Y	Y				

Inhibition assay, Y: inhibitor; ATPase activation, Y: activator; efflux, Y: effluxed compound.

**Table 10:** Molecular descriptors calculated by PaDEL.

Descriptor Type	Descriptor ID	Class
AcidicGroupCount	nAcid	2D
ALOGP	ALogP, ALogP2, AMR	2D
APol	apol	2D
Aromatic atoms count	naAromAtom	2D
Aromatic bonds count	nAromBond	2D
Atom count	nAtom, nHeavyAtom, nH, nB, nC, nN, nO, nS, nP, nF, nCl, nBr, nI	2D
BasicGroupCount	nBase	2D
BondCount	nBonds, nBonds2, nBondsS, nBondsS2, nBondsS3, nBondsD, nBondsD2, nBondsT, nBondsQ	2D
BPol	bpol	2D
Carbon types	C1SP1, C2SP1, C1SP2, C2SP2, C3SP2, C1SP3, C2SP3, C3SP3, C4SP3	2D
HBondAcceptorCount	nHBAcc, nHBAcc2, nHBAcc3, nHBAcc_Lipinski	2D
HBondDonorCount	nHBDon, nHBDon_Lipinski	2D
LargestChain	nAtomLC	2D
LargestPiSystem	nAtomP	2D
LongestAliphaticChain	nAtomLAC	2D
MannholdLogP	MLogP	2D
McGowanVolume	McGowan_Volume	2D
MLFER	MLFER_A, MLFER_BH, MLFER_BO, MLFER_S, MLFER_E, MLFER_L	2D
Ring count	nRing, n3Ring, n4Ring, n5Ring, n6Ring, n7Ring, n8Ring, n9Ring, n10Ring, n11Ring, n12Ring, nG12Ring, nFRing, nF4Ring, nF5Ring, nF6Ring, nF7Ring, nF8Ring, nF9Ring, nF10Ring, nF11Ring, nF12Ring, nFG12Ring, nTRing, nT4Ring, nT5Ring, nT6Ring, nT7Ring, nT8Ring, nT9Ring, nT10Ring, nT11Ring, nT12Ring, nTG12Ring	2D
Rotatable bonds count	nRotB	2D
Rule of five	LipinskiFailures	2D
Topological polar surface area	TopoPSA	2D
van der Waals volume	VABC	2D
Weight	MW	2D
XLogP	XLogP	2D
Charged partial surface area	PPSA-1, PPSA-2, PPSA-3, PNSA-1, PNSA-2, PNSA-3, DPSA-1, DPSA-2, DPSA-3, FPSA-1, FPSA-2, FPSA-3, FNSA-1, FNSA-2, FNSA-3, WPSA-1, WPSA-2, WPSA-3, WNSA-1, WNSA-2, WNSA-3, RPCG, RNCG, RPCS, RNCS, THSA, TPSA, RHSA, RPSA	3D
Moment of inertia	MOMI-X, MOMI-Y, MOMI-Z, MOMI-XY, MOMI-XZ, MOMI-YZ, MOMI-R	3D
Pubchem fingerprint	Hierarchal element counts Rings in a canonic Extended Smallest Set of Smallest Rings (ESSSR) ring set Simple atom pairs Simple atom nearest neighbours Detailed atom neighbourhoods Simple SMARTS patterns Complex SMARTS patterns	fingerprint
Substructure fingerprint	-	fingerprint
Substructure fingerprint count	-	

**Table 11:** Selected test sets for inhibition, ATPase activation and monolayer efflux experiments.

Inhibition	ATPase activation	Monolayer efflux
Vinblastine	Dipyridamole	Vinblastine
Terfenadine	Vinblastine	Taxol
Ritonavir	Taxol	Ritonavir
Loratadine	Ritonavir	Clarythromycin
Monensin	Clarithromycin	Indinavir
Reserpine	Monensin	Emetine
Nelfinavir	Amprenavir	Dipyridamole
Dipyridamole	Reserpine	Monensin
Ketoconazole	Trimethoprim	Reserpine
Loperamide	Prazosin	Colchicine
Vincristine	Doxorubicin	Itraconazole
Taxol	Vincristine	Verapamil
Vinorelbine	Mitoxantrone	Nicardipine
Clarithromycin	Etoposide	Yohimbine
Itraconazole	Methotrexate	Chlorpromazine
Etoposide	Puromycin	Midazolam
Daunorubicin	Vinorelbine	Nifedipine
Mitoxantrone	Triamterene	Methotrexate
Hoechst 33342	Mannitol	Testosterone
Emetine	Cimetidine	Practolol
