# Supplementary material for: Development of Classification Models for Identifying “True” P-glycoprotein (P-gp) Inhibitors Through Inhibition, ATPase Activation and Monolayer Efflux Assays
Source: Int J Mol Sci. 2012 Jun 7;13(6):6924–43. doi: 10.3390/ijms13066924 (PMC3397504; doi:10.3390/ijms13066924)
Supplement: Supplementary file 1 [file ijms-13-06924-s001.pdf]

Development of Classification Models for Identifying "True" P-glycoprotein (P-gp) Inhibitors Through Inhibition, ATPase Activation and Monolayer Efflux Assays

Supplementary Information

Figure S1. Graphical representation of the best performing decision trees for the inhibition experiment. Two classes were used: inhibitors (Y) and non-inhibitors compounds (N). Molecular descriptors and their corresponding decision criteria are reported in rectangles. The number of classified compounds for Y/N are reported between brackets. The arrows at the bottom right show the direction of the branches. See the text for an explanation of the descriptors.

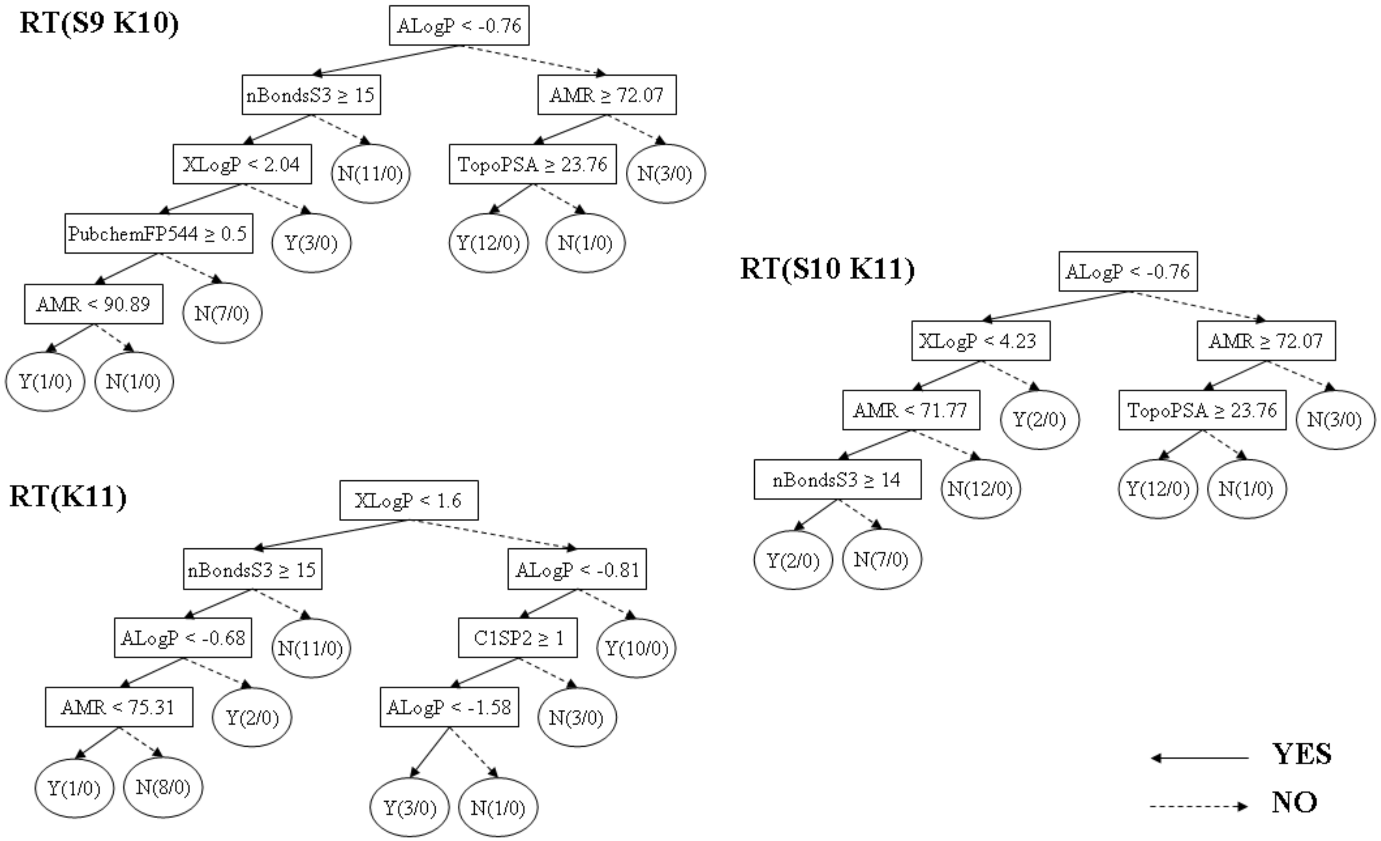

Figure S2. Graphical representation of the best performing decision trees for the ATP-ase experiment. Two classes were used: ATP-ase activators (Y) and non-activators (N). Molecular descriptors and their corresponding decision criteria are reported in rectangles. The number of classified compounds for Y/N are reported between brackets. The arrows at the bottom right show the direction of the branches. See the text for an explanation of the descriptors.

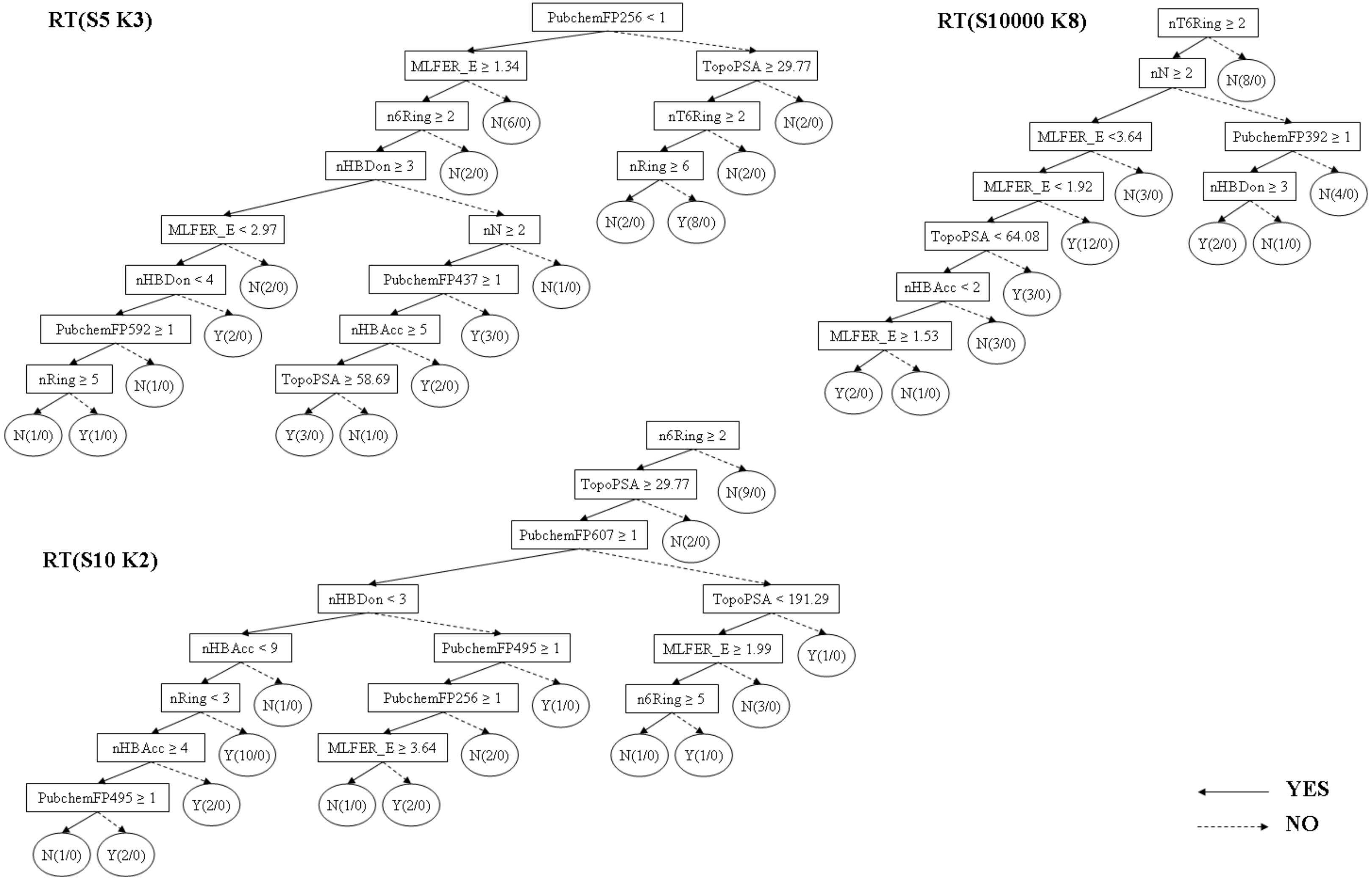

Figure S3. Graphical representation of the best performing decision trees for the monolayer efflux experiment. Two classes were used: effluxed (Y) and non-effluxed compounds (N). Molecular descriptors and their corresponding decision criteria are reported in rectangles. The number of classified compounds for Y/N are reported between brackets. The arrows at the bottom right show the direction of the branches. See the text for an explanation of the descriptors.

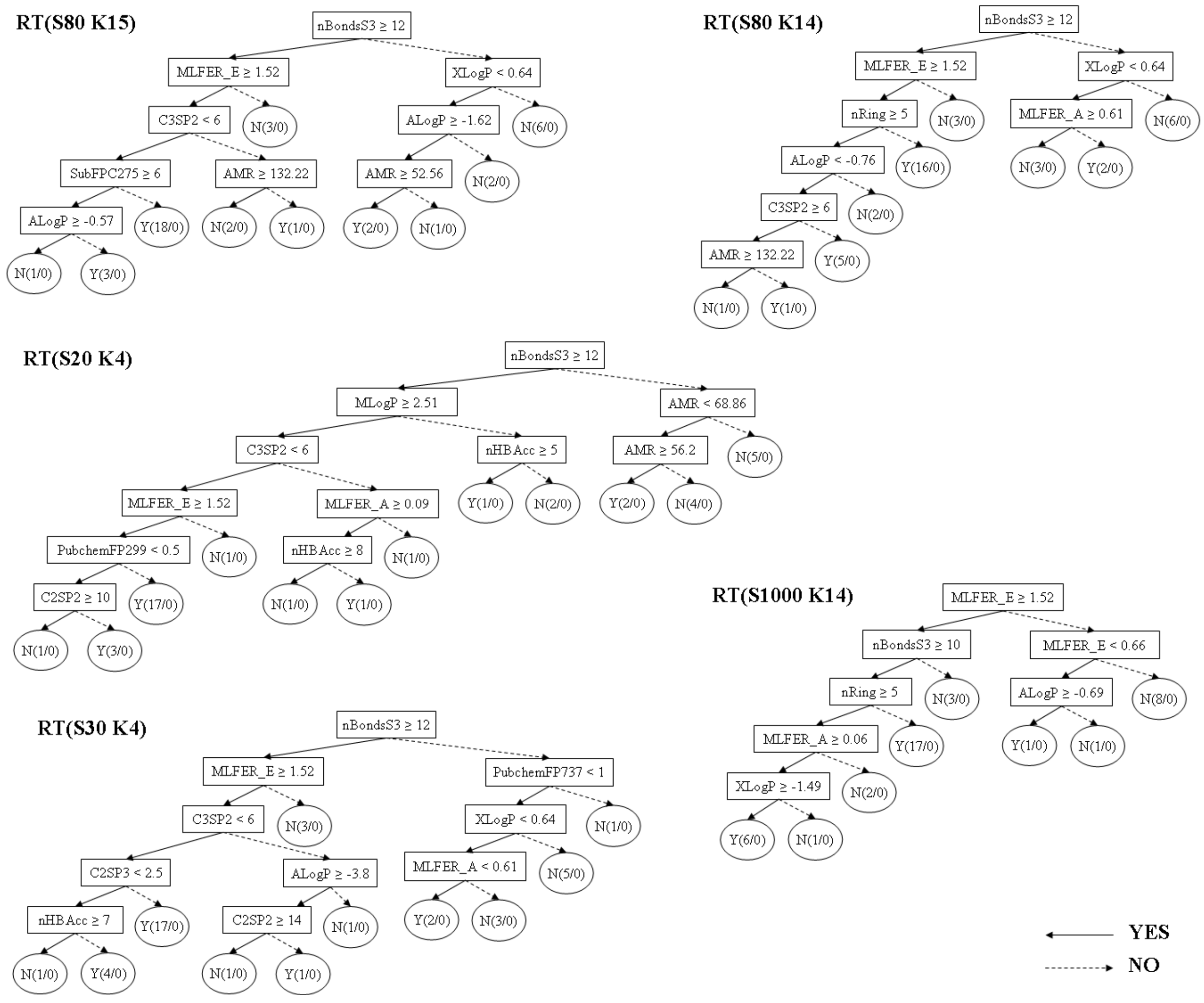

© 2012 by the authors; licensee MDPI, Basel, Switzerland. This article is an open access article distributed under the terms and conditions of the Creative Commons Attribution license (http://creativecommons.org/licenses/by/3.0/).
